# Examining Additive and Synergistic Relations Between Preschool Self-Regulation and Executive Function Skills: Predictions to Academic Outcomes

**DOI:** 10.3389/fpsyg.2021.721282

**Published:** 2021-10-28

**Authors:** Jennifer K. Finders, Robert J. Duncan, Irem Korucu, Lindsey B. Bryant, David J. Purpura, Sara A. Schmitt

**Affiliations:** ^1^Department of Human Development and Family Studies, Purdue University, West Lafayette, IN, United States; ^2^Child Study Center, Yale University, New Haven, CT, United States

**Keywords:** self-regulation, executive function, academic outcomes, teacher ratings, direct assessments, school readiness, preschool

## Abstract

In the present study, we examined the extent to which teacher-rated self-regulation and directly assessed executive function skills were independently, additively, or synergistically related to academic achievement during the transition to kindergarten. The sample included 126 children (42% female; *M*_age_ = 4.73 years) from families with low incomes who participated in a larger evaluation of state-funded preschool. Regression models with children nested in their respective preschool classrooms investigated main effects and moderated effects of teacher-rated self-regulation skills manifested in preschool classroom behaviors and cognitive executive function skills assessed through direct assessments on math, literacy, and vocabulary in the spring of preschool and in the fall of kindergarten. Results revealed independent but not additive relations between executive function and math in the spring of preschool and self-regulation and literacy in the fall of kindergarten. One significant interaction emerged providing evidence for synergistic relations between teacher-rated self-regulation and directly assessed executive function for literacy at both timepoints across the transition to kindergarten. Implications for policy and practice are discussed.

## Introduction

Self-regulation in early childhood involves top-down cognitive processes, including executive function, and bottom-up automatic regulatory processes, such as emotion regulation (Blair and Ursache, 2011). Because of its multidimensional nature, the field has wrestled with understanding the conceptual and theoretical structure of self-regulation as it relates to other similar terms and skills (Jones et al., 2016; Morrison and Grammer, 2016). This is especially prevalent in lines of research that consider the overlap and distinction with executive function (e.g., Eisenberg and Zhou, 2016; Nigg, 2017). Most researchers argue that self-regulation comprises a diverse set of abilities including executive function processes and social and emotional competencies (Hofmann et al., 2012). However, others have adopted the perspective that self-regulation and executive function represent different underlying constructs (Toplak et al., 2013). These assumptions have implications for selecting measures that are intended to capture these skills in certain contexts and for specifying statistical methods that explore associations with key indicators of school readiness. Studies have demonstrated that teacher-rated self-regulation and directly assessed executive functions independently and often additively relate to academic achievement when included simultaneously in a model (Schmitt et al., 2014; Fuhs et al., 2015; Duncan et al., 2017; Finders et al., 2021). This work generally finds that behavioral self-regulation and cognitive executive function skills both predict math and literacy in a single model, insinuating that they may tap into separate skillsets when measured in a discrete manner. Although this literature is expansive, less research has investigated whether these skills function in combination to support subsequent academic performance (Mägi et al., 2016). Existing empirical work offers two possibilities for the nature by which self-regulation and executive function may synergistically influence academic achievement. Strengths in one domain may compensate for deficits in the other (Hernández et al., 2018), or conversely, strengths in one domain may complement strengths in the other (Litkowski et al., 2020). In the present study, we examine the extent to which teacher-rated self-regulation skills manifested in preschool classroom behaviors and cognitive executive function skills assessed through direct assessments exhibit independent, additive, or synergistic relations in predicting academic outcomes during the transition to kindergarten.

### Conceptualization of Self-Regulation and Executive Function

Interest in early self-regulation and executive function has stemmed from research demonstrating their predictive links to a host of later outcomes, including academic achievement, educational attainment, and health and well-being (Moffitt et al., 2011; McClelland et al., 2013; Robson et al., 2020). Historically, the terms self-regulation and executive function have been applied interchangeably to represent similar phenomena in lines of work emerging from unique disciplines (Liew, 2012; Zhou et al., 2012; Eisenberg and Zhou, 2016; Malanchini et al., 2019). Although there is no universal definition, self-regulation is typically regarded as an umbrella term for children’s ability to utilize cognitive, behavioral, and emotional strategies to regulate their thoughts and actions while working toward a goal (McClelland et al., 2014). Executive function refers to a set of higher-order cognitive processes, including working memory, inhibitory control, and attentional flexibility, that are essential for adapting to complex and shifting environments (Miyake et al., 2000b; Diamond, 2013). These conceptualizations render self-regulation and executive function skills as separate but interrelated skillsets in childhood.

Most researchers in the field agree that executive function skills are a critical component of broader self-regulatory abilities (Rueda et al., 2005; Hofmann et al., 2012; Liew, 2012; McClelland et al., 2015). For example, children must call upon their executive function processes to engage in self-regulated interactions with peers in a classroom setting (Brock et al., 2009; Slot et al., 2017). However, executive function skills can be exercised outside of self-regulation in purely cognitive situations, for example, while working on a mathematics task (Nigg, 2017). Further, certain contexts may require children to utilize self-regulation to navigate social and emotional dynamics, beyond just engaging their executive function abilities (Blair and Razza, 2007; McClelland et al., 2007). Although progress has been made to visually map self-regulation, executive function, and related terms to illustrate their similarities and differences (Jones et al., 2016), the absence of a common framework for understanding the connection between these skills has led to “conceptual clutter” and “measurement mayhem” (Morrison and Grammer, 2016). Therefore, studies that examine the co-occurrence of both constructs have implications for improving measurement and informing interventions and practice.

### Measurement of Self-Regulation and Executive Function

The lack of precision in defining self-regulation and executive function is also reflected in measurement imperfections referred to as “task impurity” (Miyake et al., 2000a). This transpires when a single measure taps into what are thought to be theoretically discrete components of executive function or self-regulation or when the same measure is administered to assess different aspects of executive function (Packwood et al., 2011; Baggetta and Alexander, 2016). Given these limitations, researchers often rely on measurement methods to help delineate between self-regulation and executive function in early childhood. Specifically, because the definition of self-regulation considers the fit between a child and their external environment, these skills may be assessed in naturalistic settings with either direct assessments or adult ratings (McClelland and Cameron, 2012). This strategy is particularly beneficial when aiming to capture the expression of self-regulation skills within demanding contexts, such as the classroom. Executive functions tend to be less context dependent and require more internal processes, which is why these skills may be assessed under constrained conditions with direct assessments, either in lab-based settings or other controlled environments (Anderson and Reidy, 2012).

There are strengths and weaknesses of different types of assessment methods. Adult reports of self-regulation are less expensive, require little training, and have greater ecological validity, but their subjectivity can lead to issues of rater bias (Isquith et al., 2013). Direct assessments of executive function skills are more objective and have the advantage of isolating specific components of executive function (e.g., attentional flexibility), but they may not capture a child’s full capacity to self-regulate under social and emotional circumstances (McCoy, 2019). Considering these tradeoffs, there is growing consensus that implementing both approaches may offer the most comprehensive understanding of children’s development (Fuhs et al., 2015). Indeed, adult reports of self-regulation [i.e., Child Behavior Rating Scale (CBRS); Bronson et al., 1990] and direct assessments of executive function (i.e., Trails-Preschool; Espy and Cwik, 2004), are only moderately correlated, indicating they provide unique information about a child’s available cognitive resources and their use of those resources in context (Tamm and Peugh, 2019). Thus, teacher ratings offer the opportunity to measure children’s ability to apply self-regulation strategies within the classroom setting, and direct assessments of executive function provide insight into the higher-order cognitive capacities that children possess independent of context (Lipsey et al., 2017).

### Associations With Academic Achievement

In recent years, there has been interest in understanding the nature in which self-regulation and executive function skills co-occur and their implications for children’s academic learning (e.g., Lin et al., 2019). This focus reflects the field’s awareness of the potential interaction between cognitive executive function skills and behavioral self-regulation abilities for children’s academic achievement (Hernández et al., 2018). However, studies that test different conceptual notions of this dynamic have yet to consider the manifestation of these skills via teacher ratings of self-regulation. This context is critical given the strong relations between self-regulation measured within early learning environments and later life outcomes (e.g., Dettmer et al., 2020). Therefore, the following sections review empirical findings stemming from inquiry that explores independent (e.g., singular), additive (e.g., simultaneous), and synergistic (e.g., interactive) associations between teacher-rated classroom self-regulation and directly assessed executive function skills to highlight the current state of knowledge on their unique and joint implications for academic achievement.

#### Independent and Additive Relations

Much empirical work has examined the extent to which directly assessed executive function skills and teacher-rated self-regulation differentially relate to academic performance. These skills are described as having *independent* effects when one or both predicts an outcome of interest in the same model. For instance, researchers have uncovered a significant relation between kindergarten executive function, measured via the Dimensional Change Card Sort (DCCS; Zelazo, 2006), but not self-regulation, as assessed by the Child Behavior Questionnaire (CBQ; Putnam and Rothbart, 2006), and third grade math and reading, suggesting executive function has an effect that is independent of the effect of self-regulation (Finders et al., 2021). The independent relation is *additive* if both skills predict an outcome of interest when simultaneously included in a model, such that the effect of two variables is equal to the sum of their individual effects (VandenBos, 2007). To illustrate, several studies have documented additive relations between self-regulation and executive function for children’s academic achievement, with stronger associations typically emerging for direct assessments of executive function (Blair and Razza, 2007; Duncan et al., 2017; Morgan et al., 2017, 2019; Howard and Vasseleu, 2020). For instance, one study found that both direct assessments of executive function, one being the DCCS, and a teacher rating of self-regulation (i.e., Cooper-Farran Behavioral Rating Scale; Cooper and Farran, 1991) predicted children’s literacy and math in preschool when included in a model together (Fuhs et al., 2015). Domain-specific relations have also been observed among studies demonstrating additive effects, indicating that direct assessments of executive function [i.e., Head-Toes-Knees-Shoulders (HTKS); McClelland et al., 2014] tend to be more strongly related to children’s math skills, and teacher ratings of self-regulation (i.e., CBRS) appear to show greater associations with children’s literacy (von Suchodoletz et al., 2013; Schmitt et al., 2014). Together, this evidence corroborates theoretical notions that teacher ratings of self-regulation and direct assessments of executive function abilities tap into different underlying skillsets, which may be singularly or simultaneously related to children’s academic skills. Further, results insinuate there may be two potential targets for interventions and programs – strengthening self-regulation skills manifested in classroom behaviors and individual executive function abilities prior to kindergarten. Thus, research supports independent and additive relations, evidenced by one or more main effects, between the indicators of these constructs with respect to academic achievement during the early years. However, the relative significance of each component seems to vary as a function of the academic domain under investigation (Robson et al., 2020).

#### Synergistic Relations

Few studies have considered the interplay between teacher-rated self-regulation and directly assessed executive function skills. Teacher-rated self-regulation and directly assessed executive function skills may work together to support academic learning in complex ways that are not yet well understood. Specifically, these skills may exhibit a *synergistic* effect on an outcome of interest that is multiplicative, such that the effect of one variable is conditional on the other variable (VandenBos, 2007). Given that the definition of self-regulation encompasses executive function and related processes, it is often assumed that children must have strong executive function skills in order to apply them within the classroom context and demonstrate self-regulation. However, a recent investigation illustrated that kindergarten children can exhibit discordance in these skills, specifically revealing subgroups of children rated highly by teachers on their self-regulation as assessed by the CBQ, but who struggle on executive function tasks like the DCCS (Litkowski et al., 2020). Similar patterns amongst related skills have also been documented, with one analysis uncovering a small proportion of children with “mixed self-regulation,” characterized by performing low on executive function assessments but having average teacher reported behavioral task persistence in the early elementary grades (Mägi et al., 2016). Further, children who displayed incongruent teacher-rated self-regulation and directly assessed executive function were more likely to perform worse on subsequent academic tasks than their peers with average or high levels of both skillsets (Litkowski et al., 2020). Therefore, the ideal match between skills that produces the highest academic results may be for children to acquire complementary levels of self-regulation and executive function in early childhood. Yet, findings from another study challenge this notion, indicating that strong inhibitory control, a component of executive function, compensated for weak directly assessed behavioral self-regulation in predicting children’s reading during the early elementary grades (Hernández et al., 2018). Notably, results that are consistent with synergistic models of skill development, evidenced by a significant interaction effect between self-regulation and executive function, likely have more nuanced implications for early intervention and program efforts depending on whether they are complementary or compensatory.

### Present Study

In the present study, we examine the extent to which teacher-rated self-regulation and directly assessed executive function skills in preschool are independently, additively, or synergistically related to academic achievement during the transition to kindergarten. The existing literature does not strongly support one hypothesis over another. Therefore, our goal is to empirically explore which of these hypotheses best fit the data to provide clarification about the nature of these skills during the transition to kindergarten. Previous research suggests that teacher-rated self-regulation and directly assessed executive function skills may be *independently* or *additively* related to math and literacy (e.g., Schmitt et al., 2014; Fuhs et al., 2015; Howard and Vasseleu, 2020; Finders et al., 2021). There is also emerging evidence that these skills may *synergistically* influence academic achievement (e.g., Mägi et al., 2016). Yet, the exact nature of this reciprocity is unknown. On the one hand, children may benefit most when they demonstrate complementary strengths in both executive function and self-regulation skills (Litkowski et al., 2020). On the other hand, having strengths in one domain may help children compensate for weaknesses in the other (Hernández et al., 2018). Therefore, if it is the case that self-regulation and executive function work synergistically to influence academic achievement, we will determine whether they complement one another or whether one compensates for the other. Finally, we anticipate that the effects of these independent, additive, or synergistic relations on children’s academic performance could change during this transitional period because self-regulation and executive function skills mastered during the preschool year may be more relevant to learning in the proximal environment, and thus, may not transfer into the distal kindergarten learning context.

## Materials and Methods

### Participants

The sample for this study was a single cohort drawn from a larger evaluation of the impacts of high-quality state-funded preschool on children’s school readiness (Schmitt et al., 2021). Participants included 126 children (42% female) across 36 preschool classrooms. The majority (63%) of children attended state-funded preschool programs located in centers or ministries. The remaining children (37%) attended other community-based programs that were either located in centers, homes, or ministries. The sample was racially and ethnically diverse and represented the broader area in the midwestern United States, with 39% of parents identifying their children as Black/African American, 29% White/Caucasian, 18% Hispanic or Latinx, and 14% Multiracial or Other. Children were at least 4 years old at the start of the preschool year (*M*_age_ = 4.73 years; *SD* = 0.32 years) and all of their family incomes were at or below 127% of the federal poverty line (*M*_monthly income_ = $1,678.29; *SD* = 965.06). All families received child care subsidies to help pay for preschool.

### Procedures

The research procedures for this study were approved by the university’s institutional review board. Parents provided written consent and children verbally assented to participate in the study. Trained research assistants administered direct assessments of literacy, vocabulary, math, and executive function to children in the fall and spring of preschool and in the fall of kindergarten at their schools. Teachers rated children’s behaviors in the classroom in the fall and spring of preschool, and parents completed a questionnaire in the fall of preschool with information on family demographics. Parents and teachers received $20 in the fall and spring of preschool as compensation for their participation.

### Measures

#### Teacher-Rated Self-Regulation

Children’s self-regulation was assessed via the self-control and cooperation subscales of the Social Skills Improvement System (SSIS; Gresham and Elliott, 1990, 2008). Teachers were asked to rate the frequency of behaviors they observed in the classroom on a 4-point scale with responses ranging from 0 = *never* to 3 = *almost always*. The self-control subscale includes six items that focus on children’s impulse control and emotion regulation. Sample items include, “stays calm when teased” and “takes criticism without getting upset.” The cooperation subscale includes seven items that focus on children’s attention and persistence. Sample items include, “follows your directions” and “ignores classmates when they are distracting.” In previous research, these scales have demonstrated strong construct validity through correlations with a direct assessment of behavioral self-regulation (e.g., the HTKS task; *r* = 0.77 self-control, *r* = 0.50 cooperation) and strong predictive validity based on relations with children’s school readiness (Sektnan et al., 2010; MacDonald et al., 2016). In this sample, Cronbach’s alpha was high at fall (α = 0.92) and spring (α = 0.90) for all 13 items. A composite score was generated by averaging all items from both subscales in the fall and spring (26 items total). Possible scores ranged from 0 to 3. A similar approach has been taken in previous research to capture learning related skills with the Social Skills Rating Scale, an older version of the SSIS, in combination with additional teacher rating scales (McClelland and Morrison, 2003).

#### Directly Assessed Executive Function

Children’s executive function was assessed via the Dimension Change Card Sort task (DCCS; Zelazo, 2006). During this 24-item assessment, children are instructed to sort cards into boxes according to different rules in three sections (i.e., six cards based on shape, six cards based on color, and six cards based on size). In the final section, children are required to sort six cards based on two complex rules which depend on whether the cards have a border or not (i.e., border cards sorted on size, no border cards sorted on color). Children receive 1 point for each correct response. Possible total scores ranged from 0 to 24. The standard DCCS has been shown to be a valid and reliable measure of attentional flexibility among preschoolers (Zelazo, 2006; Zelazo et al., 2013). Moreover, previous research using the version of the DCCS with the border section among this age group has found correlations between *r* = 0.28 (fall of kindergarten) and *r* = 0.36 (fall of preschool) with other direct assessments of executive function (i.e., HTKS; McClelland et al., 2014). In this sample, Cronbach’s alpha was high at fall (α = 0.88) and spring (α = 0.89) for all DCCS items. A composite score was generated averaging the total scores from fall and spring.

#### Academic Achievement

##### Mathematics

Children’s early math skills were assessed via the Woodcock Johnson IV Tests of Achievement – Applied Problems subtest (Schrank et al., 2014). This 56-item standardized task requires children to respond verbally or by pointing to their answer in a testing booklet to questions that assess counting, addition, subtraction, and story problems. For example, children are asked, “how many dogs are there?” Children receive 1 point for each correct response. The task ends when children respond incorrectly to 5 questions in a row. Raw scores were used in analyses with a possible range from 0 to 56. The Applied Problems subtest has demonstrated strong reliability (Villarreal, 2015).

##### Literacy

Children’s literacy skills were assessed via the Woodcock Johnson IV Tests of Achievement – Letter-Word Identification subtest (Schrank et al., 2014). This 76-item standardized task requires children to respond verbally or by pointing to their answer in a testing booklet to questions that assess letter identification and word reading. For instance, children are shown words on a page and asked, “point to the word sun” or “what is this word?” Children receive 1 point for each correct response. The task ends when children respond incorrectly to 6 items in a row. Raw scores were used in analyses with a possible range from 0 to 76. The Letter-Word Identification subtest has demonstrated strong reliability (Villarreal, 2015).

##### Vocabulary

Children’s receptive vocabulary skills were assessed via the Peabody Picture Vocabulary Test – IV (PPVT; Dunn and Dunn, 2007). In this task, children are instructed to point to the picture in a testing booklet that best represents the verbal cue given by the assessor. For instance, they may be shown four pictures and asked, “point to dancing,” or “point to globe.” Children receive 1 point for each correct response. The assessor begins the task at the section that is appropriate for a child’s age (e.g., 4 years-old). A basal must first be established where children have to respond correctly to 11 out of 12 items in a set before moving forward through the task. The task ends when children respond incorrectly to 8 items or more in a set. Total raw scores were used in analyses with a possible range of 0–228. The PPVT – IV has been shown to be reliable and valid (Bauer and Zelazo, 2013).

#### Covariates

We accounted for several background characteristics that have been linked to children’s school readiness outcomes, including child age, sex, race/ethnicity, and family monthly income (Wanless et al., 2011; Reardon and Portilla, 2016). We also included a variable for group (state-funded preschool or comparison) to control for different learning experiences during the preschool year.

### Analytic Strategy

The analyses proceeded in two steps. First, two regression models were executed in Stata 16.0 (Stata Corp, 2019) using the structural equation modeling (SEM) command to examine independent and additive relations (i.e., main effects) of teacher-rated self-regulation and directly assessed executive function on children’s math, literacy, and vocabulary outcomes in the spring of preschool and in the fall of kindergarten. Specifically, teacher-rated self-regulation and directly assessed executive function were entered as simultaneous predictors of children’s academic outcomes in the spring of preschool, along with the battery of covariates. The same model was run for outcomes in the fall of kindergarten. Error terms for math, literacy, and vocabulary outcomes were allowed to covary in both models. Then, two additional regression models were run to explore synergistic relations (i.e., moderated effects) between teacher-rated self-regulation and directly assessed executive function skills on academic outcomes in the spring of preschool and in the fall of kindergarten. These were essentially the same models as specified and described above, with the addition of an interaction between teacher-rated self-regulation and directly assessed executive function. Error terms for math, literacy, and vocabulary outcomes were allowed to covary in these two models as well. The SEM command in Stata can be utilized with observed variables and has the advantage over typical regression of allowing for full information maximum likelihood (FIML) estimation and correlated error terms of the outcomes. All analyses controlled for prior academic skills in the fall of preschool (i.e., math, literacy, and vocabulary), child sex, age, race/ethnicity, preschool group, and family monthly income. The variables for self-regulation, executive function, and income were mean centered prior to generating the interactions, and standard errors were clustered at the preschool classroom-level to account for the nested structure of the data. We present standardized regression coefficients from all models, which can be interpreted as effect sizes.

#### Missing Data

There was a small amount of missing data on direct assessments of executive function in the fall (5%) and spring (11%) of preschool and teacher ratings of self-regulation in preschool (2%). Few children were also missing data on direct assessments of academic skills measured in the fall of preschool (3–5%), spring of preschool (10–13%), and fall of kindergarten (10%). Therefore, FIML was utilized to approximate values of missing data. This method is preferred over listwise deletion because it produces less biased estimates (Acock, 2012).

## Results

### Descriptive Statistics and Correlations

Descriptive statistics for all study variables are in Table 1. Bivariate correlations for primary study variables are presented in Table 2. The correlation between teacher ratings of self-regulation and the direct assessment of individual executive function in preschool was not statistically significant (*r* = 0.17, *p* = 0.055). Preschool executive function was significantly and positively correlated with all academic outcomes in the fall of preschool (*r*s = 0.41–0.50, *p*s < 0.001), spring of preschool (*r*s = 0.41–0.56, *p*s < 0.001), and fall of kindergarten (*r*s = 0.39–0.76, *p*s < 0.001). However, preschool self-regulation was only significantly and positively correlated with fall preschool literacy (*r* = 0.19, *p* = 0.039), fall kindergarten literacy (*r* = 0.22, *p* = 0.017), and fall kindergarten math (*r* = 0.22, *p* = 0.018). Math, literacy, and vocabulary were significantly and positively correlated with each other in the fall of preschool (*r*s = 0.36–0.61, *p*s < 0.001), spring of preschool (*r*s = 0.39–0.54, *p*s < 0.001), and fall of kindergarten (*r*s = 0.48–0.53, *p*s < 0.001).

**TABLE 1:** Descriptive statistics for sample demographics and primary study variables (*n* = 126).

**Variable**	**N**	**Mean**	**SD**	**Range**
**Preschool**				
Fall math	120	9.13	3.51	1–16
Fall literacy	121	6.21	3.62	0–13
Fall vocabulary	122	67.18	19.28	16–114
Self-regulation	124	1.81	0.55	0–3
Executive function	124	11.69	5.27	4–21.5
Spring math	113	10.74	3.35	2–21
Spring literacy	113	7.94	4.95	0–38
Spring vocabulary	110	74.90	20.93	21–122
**Kindergarten**				
Fall math	113	13.44	3.46	4–22
Fall literacy	113	12.55	5.72	3–45
Fall vocabulary	113	88.88	18.38	39–127
**Covariates**				
Age (in years)	124	4.73	0.32	3.85–5.27
Monthly income	126	1,678.29	965.06	0–6,257.77
Female	126	0.42	0.50	0–1
Group	126	0.63	0.48	0–1
**Race/Ethnicity**				
White/Caucasian	36	0.29	0.46	0–1
Black/African American	48	0.39	0.49	0–1
Latinx	22	0.18	0.38	0–1
Mixed/Other	17	0.14	0.35	0–1

*Preschool self-regulation and executive function were averaged across fall and spring of preschool.*

**TABLE 2:** Correlations between primary study variables (*n* = 126).

**Variables**	**1**	**2**	**3**	**4**	**5**	**6**	**7**	**8**	**9**	**10**
1. Fall preschool math	–									
2. Fall preschool literacy	0.36***	–								
3. Fall preschool vocabulary	0.61***	0.42***	–							
4. Preschool self-regulation	0.14	0.19*	0.11	–						
5. Preschool executive function	0.47***	0.41***	0.50***	0.17	–					
6. Spring preschool math	0.59***	0.48***	0.44***	0.18	0.56***	–				
7. Spring preschool literacy	0.32***	0.76***	0.34***	0.18	0.41***	0.47***	–			
8. Spring preschool vocabulary	0.61***	0.41***	0.77***	0.14	0.53***	0.54***	0.39***	–		
9. Fall kindergarten math	0.58***	0.34***	0.42***	0.22*	0.39***	0.61***	0.38***	0.52***	–	
10. Fall kindergarten literacy	0.35***	0.59***	0.41***	0.22*	0.76***	0.41***	0.50***	0.46***	0.53***	–
11. Fall kindergarten vocabulary	0.51***	0.42***	0.78***	0.12	0.46***	0.57***	0.36***	0.73***	0.50***	0.48***

Preschool self-regulation and executive function were averaged across fall and spring of preschool; *p < 0.05 and ***p < 0.001.

### Independent and Additive Models

Results from regression models examining main effects of teacher-rated self-regulation and direct assessments of executive function provided support for independent but not additive relations between these preschool skills and academic outcomes during the transition to kindergarten. Specifically, directly assessed executive function abilities predicted children’s math in the spring of preschool (*B* = 0.29, *SE* = 0.06, *p* < 0.001), after controlling for initial academic skills and the battery of child and family covariates (Table 3). However, this association was not statistically significant in the fall of kindergarten. Further, teacher-rated self-regulation predicted children’s literacy in the fall of kindergarten (*B* = 0.14, *SE* = 0.06, *p* = 0.031), but this association was not significant in the spring of preschool (Table 4). Neither directly assessed executive function abilities nor teacher-rated self-regulation skills were significantly related to children’s vocabulary at either timepoint (Table 5). Further, because teacher-rated self-regulation and directly assessed executive function skills were not simultaneously related to any outcome, these associations did not constitute evidence of additive relations. In terms of the academic covariates in the fall of preschool, both math (*B* = 0.35, *SE* = 0.07, *p* < 0.001) and literacy (*B* = 0.28, *SE* = 0.07, *p* < 0.001) predicted children’s subsequent math skills in the spring of preschool, but only math remained a significant predictor of math in the fall of kindergarten (*B* = 0.45, *SE* = 0.08, *p* < 0.001). Fall of preschool literacy was the only academic skill that predicted literacy in the spring of preschool (*B* = 0.70, *SE* = 0.08, *p* < 0.001) and in the fall of kindergarten (*B* = 0.45, *SE* = 0.05, *p* < 0.001). Fall of preschool math predicted vocabulary in the spring of preschool (*B* = 0.17, *SE* = 0.05, *p* = 0.001), but not in the fall of kindergarten. Finally, fall of preschool vocabulary predicted subsequent vocabulary skills in the spring of preschool (*B* = 0.58, *SE* = 0.07, *p* < 0.001) and in the fall of kindergarten (*B* = 0.66, *SE* = 0.07, *p* < 0.001).

**TABLE 3:** Model estimates for additive and synergistic effects of preschool self-regulation and executive function on math outcomes.

	**Spring preschool**		**Fall kindergarten**	
	**Additive**	**Synergistic**	**Additive**	**Synergistic**
	**B (SE)**	**B (SE)**	**B (SE)**	**B (SE)**
**Covariates**				
Age (in years)	−0.05(0.05)	−0.05(0.05)	−0.01(0.08)	−0.01(0.08)
Monthly income	−0.04(0.08)	−0.04(0.08)	−0.10(0.05)*	−0.10(0.05)
Female	−0.14(0.05)**	−0.14(0.05)**	−0.06(0.07)	−0.06(0.07)
Group	0.02 (0.08)	0.02 (0.08)	−0.01(0.07)	−0.01(0.07)
**Race/Ethnicity**				
White (reference)				
Black/African American	0.01 (0.07)	0.01 (0.07)	−0.01(0.09)	−0.01(0.09)
Hispanic/Latinx	0.07 (0.10)	0.07 (0.10)	0.07 (0.12)	0.07 (0.12)
Mixed/Other	0.10 (0.07)	0.10 (0.07)	0.02 (0.08)	0.02 (0.08)
**Preschool skills**				
Fall math	0.35(0.07)***	0.35(0.07)***	0.45(0.08)***	0.45(0.09)***
Fall literacy	0.28(0.07)***	0.28(0.07)***	0.12 (0.09)	0.12 (0.09)
Fall vocabulary	0.03 (0.10)	0.03 (0.10)	0.03 (0.10)	0.03 (0.10)
Self-regulation	0.05 (0.09)	0.05 (0.10)	0.12 (0.07)	0.12 (0.07)
Executive function	0.29(0.06)***	0.29(0.06)***	0.13 (0.10)	0.13 (0.10)
Self-regulation × Executive Function		−0.01(0.08)		−0.01(0.08)
*R* ^2^	0.54	0.54	0.41	0.41

Preschool self-regulation and executive function were averaged across fall and spring of preschool; *p < 0.05, **p < 0.01, and ***p < 0.001; Standardized beta coefficients shown; Additive model is a test of main effects; Synergistic model includes the interaction effect.

**TABLE 4:** Model estimates for additive and synergistic effects of preschool self-regulation and executive function on literacy outcomes.

	**Spring preschool**		**Fall kindergarten**	
	**Additive**	**Synergistic**	**Additive**	**Synergistic**
	**B (SE)**	**B (SE)**	**B (SE)**	**B (SE)**
**Covariates**				
Age (in years)	0.02 (0.07)	0.01 (0.06)	0.01 (0.07)	−0.02(0.07)
Monthly income	0.05 (0.05)	0.05 (0.05)	0.04 (0.05)	0.02 (0.04)
Female	0.07 (0.04)	0.07 (0.04)	−0.07(0.09)	−0.09(0.08)
Group	0.04 (0.04)	0.03 (0.04)	0.14(0.05)**	0.13(0.05)**
**Race/Ethnicity**				
White (reference)				
Black/African American	−0.08(0.07)	−0.10(0.07)	−0.05(0.09)	−0.07(0.09)
Hispanic/Latinx	−0.04(0.07)	−0.05(0.08)	−0.05(0.08)	−0.07(0.09)
Mixed/Other	0.00 (0.06)	−0.01(0.06)	−0.04(0.06)	−0.06(0.06)
**Preschool skills**				
Fall math	0.04 (0.08)	0.04 (0.08)	0.05 (0.07)	0.02 (0.06)
Fall literacy	0.70(0.08)***	0.69(0.09)***	0.45(0.05)***	0.43(0.06)***
Fall vocabulary	−0.01(0.06)	−0.00(0.06)	0.10 (0.11)	0.11 (0.10)
Self-regulation	0.01 (0.05)	0.03 (0.06)	0.14(0.06)*	0.18(0.07)*
Executive function	0.10 (0.09)	0.10 (0.08)	0.13 (0.11)	0.14 (0.10)
Self-regulation × Executive Function		0.12(0.06)*		0.21(0.06)**
*R* ^2^	0.62	0.64	0.46	0.50

Preschool self-regulation and executive function were averaged across fall and spring of preschool; *p < 0.05, **p < 0.01, and ***p < 0.001; Standardized beta coefficients shown; Additive model is a test of main effects; Synergistic model includes the interaction effect.

**TABLE 5:** Model estimates for additive and synergistic effects of preschool self-regulation and executive function on vocabulary outcomes.

	**Spring preschool**		**Fall kindergarten**	
	**Additive**	**Synergistic**	**Additive**	**Synergistic**
	**B (SE)**	**B (SE)**	**B (SE)**	**B (SE)**
**Covariates**				
Age (in years)	0.08(0.05)	0.09(0.05)	0.04(0.06)	0.05(0.06)
Monthly income	0.05(0.05)	0.05(0.05)	−0.09(0.04)*	−0.09(0.04)*
Female	0.06(0.04)	0.06(0.04)	−0.11(0.05)*	−0.11(0.05)*
Group	−0.03(0.04)	−0.02(0.04)	0.04(0.06)	0.04(0.06)
**Race/Ethnicity**				
White (reference)				
Black/African American	0.11(0.06)	0.12(0.06)*	0.01(0.07)	0.01(0.07)
Hispanic/Latinx	0.01(0.07)	0.02(0.07)	0.04(0.07)	0.04(0.07)
Mixed/Other	−0.04(0.07)	−0.04(0.07)	−0.04(0.05)	−0.04(0.05)
**Preschool skills**				
Fall math	0.17(0.05)**	0.17(0.05)**	0.01(0.04)	0.02(0.04)
Fall literacy	0.08(0.05)	0.08(0.05)	0.08(0.06)	0.09(0.06)
Fall vocabulary	0.58(0.07)***	0.58(0.07)***	0.66(0.07)***	0.66(0.07)***
Self-regulation	0.03(0.07)	0.02(0.07)	0.03(0.07)	0.02(0.06)
Executive function	0.09(0.07)	0.09(0.07)	0.12(0.07)	0.12(0.07)
Self-regulation × Executive Function		−0.05(0.04)		−0.05(0.07)
*R* ^2^	0.70	0.71	0.67	0.67

Preschool self-regulation and executive function were averaged across fall and spring of preschool; *p < 0.05, **p < 0.01, and ***p < 0.001; Standardized beta coefficients shown; Additive model is a test of main effects; Synergistic model includes the interaction effect.

### Synergistic Models

**FIGURE 1:**
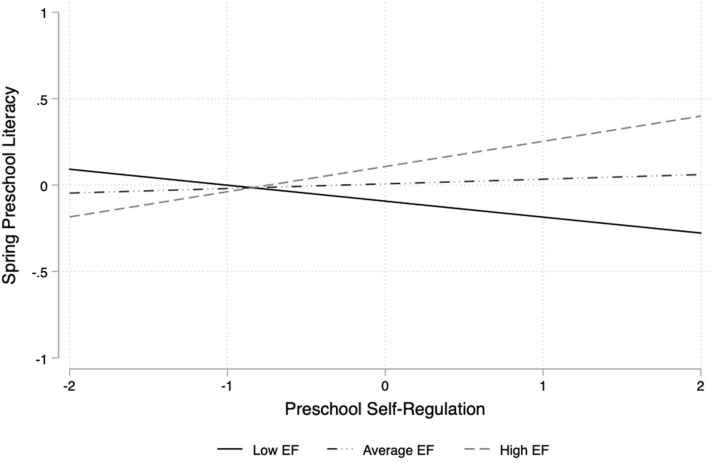
Interaction between teacher-rated self-regulation and direct assessment of executive function for children’s spring of preschool literacy skills. Note. EF = Executive Function; Executive Function and Self-Regulation are mean-centered and standardized; Low EF = 1 SD below the mean, Average EF = mean, High EF = 1 SD above the mean.

**FIGURE 2:**
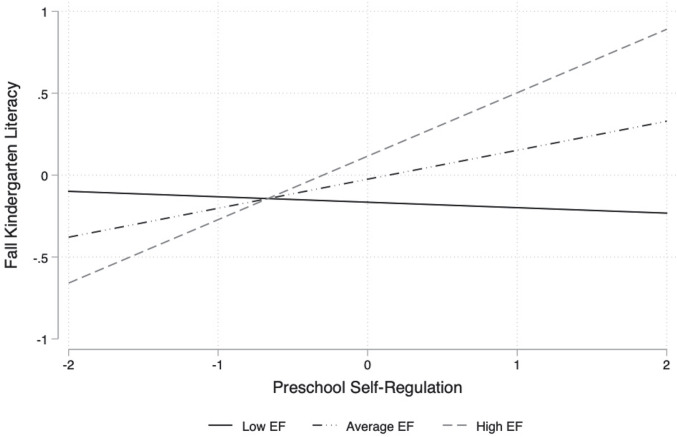
Interaction between teacher-rated self-regulation and direct assessment of executive function for children’s fall of kindergarten literacy skills. Note. EF = Executive Function; Executive Function and Self-Regulation are mean-centered and standardized; Low EF = 1 SD below the mean, Average EF = mean, High EF = 1 SD above the mean.

Results from the regression models examining moderated effects revealed synergistic relations between teacher-rated self-regulation and direct assessments of executive function in preschool for children’s literacy (Table 4). Specifically, two statistically significant interactions indicated that the positive association between teacher-rated self-regulation and literacy achievement was stronger among children who also performed highly on direct assessments of executive function in the spring of preschool (*B* = 0.12, *SE* = 0.06, *p* = 0.036) and in the fall of kindergarten (*B* = 0.21, *SE* = 0.06, *p* = 0.001). Graphical illustrations of these interactions are presented in Figures 1, 2. Notably, effects grew stronger from spring of preschool to fall of kindergarten, despite controlling for initial academic skills and the host of child and family covariates. However, there were no other statistically significant interactions between teacher-rated self-regulation and directly assessed executive function skills for children’s math (Table 3) or vocabulary (Table 5) during the transition to kindergarten. In terms of the academic covariates in the fall of preschool, the same pattern of results emerged for the synergistic models as the additive models. Both math (*B* = 0.35, *SE* = 0.07, *p* < 0.001) and literacy (*B* = 0.28, *SE* = 0.07, *p* < 0.001) predicted children’s subsequent math skills in the spring of preschool, but only math remained a significant predictor of math in the fall of kindergarten (*B* = 0.45, *SE* = 0.09, *p* < 0.001). Fall of preschool literacy was the only academic skill that predicted literacy in the spring of preschool (*B* = 0.69, *SE* = 0.09, *p* < 0.001) and in the fall of kindergarten (*B* = 0.43, *SE* = 0.06, *p* < 0.001). Fall of preschool math predicted vocabulary in the spring of preschool (*B* = 0.17, *SE* = 0.05, *p* = 0.001), but not in the fall of kindergarten. Finally, fall of preschool vocabulary predicted subsequent vocabulary skills in the spring of preschool (*B* = 0.58, *SE* = 0.07, *p* < 0.001) and in the fall of kindergarten (*B* = 0.66, *SE* = 0.07, *p* < 0.001).

## Discussion

The goal of the present study was to examine the extent to which self-regulation and executive function skills were additively or synergistically related to academic achievement during the transition to kindergarten. By leveraging teacher ratings of self-regulation and direct assessments of executive function abilities, we aimed to clarify the nature in which these related but unique skillsets predicted children’s math, literacy, and vocabulary outcomes – both on their own and in combination with one another. Overall, results revealed independent associations between directly assessed executive function and math and teacher-rated self-regulation and literacy that were timepoint specific. Further, findings provided evidence of synergistic relations between teacher-rated self-regulation and directly assessed executive function skills for children’s literacy that persisted across the transition to kindergarten. Together, this study contributes to our understanding of how these skills may work individually and collectively in complex ways to enhance children’s academic performance during the transition to kindergarten.

With regards to additive associations, previous research has demonstrated that direct assessments of executive function and teacher ratings of self-regulation simultaneously predict children’s early academic skills when included in models together (e.g., Blair and Razza, 2007; Fuhs et al., 2015; Morgan et al., 2017). We expected that the same may occur in this study. However, our results ran contrary to this previous research, indicating that teacher-rated self-regulation and direct assessments of executive function skills both did not predict the same outcomes. Instead, these skills exhibited independent relations to academic skills that were timepoint specific. Specifically, executive function was indicative of spring of preschool math and self-regulation was indicative of fall of kindergarten literacy performance. This pattern of findings is consistent with one study that found relations between direct assessments of executive function and math and literacy in third grade after controlling for teacher-rated self-regulation (Finders et al., 2021). Moreover, results align with research documenting stronger domain-specific relations between executive function and math and behavioral self-regulation and literacy (e.g., von Suchodoletz et al., 2013; Schmitt et al., 2014). Further, it confirms that teacher-rated self-regulation displayed through classroom behaviors and direct assessments of cognitive executive function abilities may tap into different skills in context that each have the unique ability to forecast children’s learning (Toplak et al., 2013; Fuhs et al., 2015; Tamm and Peugh, 2019).

In terms of synergistic relations, we adopted an exploratory approach to hypothesis testing as previous research has provided mixed support for the nature of these associations (Hernández et al., 2018; Litkowski et al., 2020). Our results were more consistent with findings from Litkowski et al. (2020), suggesting that complementary high levels of teacher-rated self-regulation and directly assessed executive function skills in preschool provided the ideal ingredients for children to achieve higher literacy scores during the transition to kindergarten. Notably, having high skills in one domain did not make up for having lower skills in another. But rather, children who exhibited a match between their cognitive resources and their ability to effectively utilize those resources within learning contexts had stronger literacy outcomes. Why these skills were dependent upon one another may be because many reading tasks require coordinating multiple mental representations, including switching between word reading to word meaning (Cartwright, 2009; Colé et al., 2014). Indeed, researchers have found strong associations between attentional flexibility and literacy in preschool among children attending Head Start (Bierman et al., 2008), and have documented the mediating role of approaches to learning in the association between attentional flexibility and school readiness (Vitiello et al., 2011). We expand on this prior work in an important way by demonstrating that these skills may augment each other rather than account for one another in terms of how they support children’s later literacy performance.

Unlike the independent associations, the effects of these synergistic relations on literacy achievement endured and grew stronger from spring of preschool to fall of kindergarten. In other words, learning to apply diverse skillsets in combination and tapping into a greater number of cognitive and behavioral resources predicted successful adjustment to classroom demands within the proximal preschool and distal kindergarten environments. These findings are consistent with prior work demonstrating an interaction between directly assessed self-regulation and executive function for children’s reading but not math in the early elementary grades (Hernández et al., 2018). One reason for this may be due to the fact that children are likely exposed to redundant literacy content in kindergarten (Cohen-Vogel et al., 2021). Therefore, having the capacity to both persist and ignore distractions at the same time as flexibly exercising attention may be critical for learning basic literacy skills, which teachers are then prone to repeat during kindergarten instruction. It is also possible that literacy skills are more malleable to the classroom environment and can be influenced by children’s learning in other domains relative to math or vocabulary skills, particularly because preschool and kindergarten teachers spend majority of their time focusing on developing reading abilities (Early et al., 2010; Bassok et al., 2016). Regardless, a critical next step will be examining the mechanisms through which teacher-rated self-regulation and directly assessed executive function create the optimal condition to support literacy development.

Finally, it should be noted that within-domain skills were consistent and strong predictors of all outcomes across time points. For vocabulary skill development, prior vocabulary was the only skill that stably explained variance in later vocabulary skills, with the relation enhancing over time (*B* = 0.58 in spring of preschool and *B* = 0.66 in fall of kindergarten). For literacy, the effect size for fall of preschool literacy (*B* = 0.69 in spring of preschool and *B* = 0.43 in fall of kindergarten) was three-to-five times the effect size for the interaction between preschool executive function and self-regulation on literacy outcomes (*B* = 0.12 at both time points). This suggests that prior letter identification and reading skills are highly indicative of later literacy performance, beyond behavioral self-regulation and cognitive executive function abilities. Alternatively, the effect size for preschool executive function on math in the spring of preschool (*B* = 0.29) was similar to the effect size for fall of preschool math (*B* = 0.35), although executive function did not remain a significant predictor of math in the fall of kindergarten. Other research has uncovered a similar pattern of results between these skills, demonstrating correlated growth between math and executive function but not between executive function and literacy (see Schmitt et al., 2017). Our study provides corroborating evidence that executive function and math are very closely related in early childhood (Nguyen et al., 2019), and therefore may share variance in explaining math abilities. Regardless, the large autoregressive estimates indicate that comprehensive approaches to intervention that target both academic skills alongside executive function and self-regulation may offer the largest benefit. For instance, several interventions have been developed that help children practice their self-regulation or executive function skills (Diamond and Lee, 2011), with many demonstrating positive effects on academic achievement as well (Pandey et al., 2018).

### Limitations and Future Directions

Although this study adds to extant literature, several limitations must be acknowledged. First and foremost, the data are correlational and therefore we cannot make any causal conclusions about the nature of the relations observed in this study. Future research should continue to examine the consequences of both skillsets in the context of preschool programs and interventions to garner more empirical support for these complex relations from rigorous experimental designs. Additionally, we included a robust set of control variables in the models, but there were factors that we could not account for that may be related to children’s skills during this period of development, such as the extent to which parents were involved in preparing their children for the transition to kindergarten (Pears et al., 2014). Further, although we view it as a strength that our sample of children were all from families with low incomes because this population tends to start formal schooling behind their more advantaged peers in key school readiness domains (Reardon and Portilla, 2016), the results may not be generalizable to children with diverse backgrounds and a range of early experiences. Thus, replicating these findings across datasets and examining potential mediators and mechanisms is a necessary future direction.

In terms of measurement, we were limited to using two subscales of the SSIS to assess teacher-rated self-regulation abilities manifested in classroom behaviors. Researchers have used the self-control and cooperation subscales in prior work to measure a similar construct of learning related skills (McClelland and Morrison, 2003). Further, these scales have demonstrated construct validity through their strong association with a commonly administered measure of behavioral self-regulation (MacDonald et al., 2016). Additionally, because of the widespread issue of task impurity, we cannot be completely confident that we are capturing an underlying construct of self-regulation (Morrison and Grammer, 2016). The same is true for our direct assessment of executive function. While the structure of executive function is an ongoing debate (Karr et al., 2018), like others, we maintain that the DCCS is an accurate representation of executive function at this age because in addition to flexibly switching between mental representations of card characteristics, children must inhibit their immediate response to sort cards by the previous dimension and remember and use different rules as they advance through the task (i.e., tapping all three dimensions of executive function; Doebel and Zelazo, 2015). Yet, future research should continue to explore these associations as researchers draw firmer conclusions about the structure of executive function in early childhood and as new tasks emerge that have the ability to better distinguish between executive components.

Finally, an issue in this study and the field at-large is that these related skills are often differentiated by the type of measurement instrument itself (i.e., direct assessment of executive function versus teacher rating of self-regulation). Researchers who wish to overcome this limitation by taking advantage of teacher-ratings of executive function, like the Behavior Rating Inventory of Executive Function, may also face the issue of task impurity if there is overlap between self-regulation and executive function constructs (Gioia et al., 2000). Nevertheless, because the assessment protocol followed in this study is common practice in the field, we have the opportunity to situate findings within the broader literature. In sum, reproducing these models with a larger battery of teacher ratings of self-regulation and direct assessments of executive function will be important for demonstrating how various modeling techniques may be specified in order to overcome some of these limitations.

## Conclusion

Overall, results from the present study contribute to the field’s understanding of the nature of relations between teacher-rated self-regulation expressed in classroom behaviors and cognitive executive functions measured by direct assessments for children’s academic achievement during the transition to kindergarten. Specifically, findings demonstrate how these critical skillsets may operate synergistically to enhance children’s literacy development, such that possessing strong self-regulation skills *and* executive function abilities in early childhood may provide children with the optimal access to resources to call upon in circumstances where they need to meet rigorous academically oriented demands. Therefore, practitioners are likely to benefit from implementing instructional approaches that comprehensively support broader self-regulation strategies and specific executive function skills, ensuring that children develop complementary levels of both skillsets in preschool. Similarly, policymakers may earn the highest return on their investment in early education programs by assessing both types of abilities in the early years as children are transitioning into the formal schooling environment. Still, the effect sizes for academic skills in the fall of preschool were similar and often stronger than those of executive function or self-regulation, indicating their significant role in children’s educational attainment. Finally, researchers should continue to focus on developing measures that can effectively capture these unique skillsets and explore statistical methods that deepen our knowledge of their complex relations over time.

## Data Availability Statement

The datasets presented in this article are not readily available because the study is still ongoing. Requests to access the datasets should be directed to SS, saraschmitt@purdue.edu.

## Ethics Statement

The studies involving human participants were reviewed and approved by Purdue University Institutional Review Board (IRB). Written informed consent to participate in this study was provided by the participants’ legal guardian/next of kin.

## Author Contributions

JF: conceptualization, data curation, investigation, methodology, visualization, and writing – original draft. RD: conceptualization, funding acquisition, project administration, formal analysis, and writing – review and editing. IK and LB: writing – review and editing. DP: conceptualization, funding acquisition, project administration, and writing – review and editing. SS: conceptualization, funding acquisition, project administration, resources, and writing – review and editing. All authors contributed to the article and approved the submitted version.

## Conflict of Interest

The authors declare that the research was conducted in the absence of any commercial or financial relationships that could be construed as a potential conflict of interest.

## Publisher’s Note

All claims expressed in this article are solely those of the authors and do not necessarily represent those of their affiliated organizations, or those of the publisher, the editors and the reviewers. Any product that may be evaluated in this article, or claim that may be made by its manufacturer, is not guaranteed or endorsed by the publisher.
